# PReS-FINAL-2016: Bone mineral status in a long-term follow up of juvenile dermatomyositis patients

**DOI:** 10.1186/1546-0096-11-S2-P29

**Published:** 2013-12-05

**Authors:** VA Kristensen, S Nielsen, T Herlin, P Mathiesen

**Affiliations:** 1 Paediatrics, University Hospital, Holbaek, Denmark; 2 Paediatric Rheumatology Unit, Rigshospitalet, University Hospital, Copenhagen, Denmark; 3 Paediatrics, University Hospital, Aarhus, Denmark

## Introduction

Juvenile dermatomyositis (JDM) is a rare autoimmune disease with the onset in childhood involving chronic inflammation of striated muscle and skin. The disease is often leading to severe disability, prolonged decreased physical activity, which together with chronic inflammatory activity, and long-term medical treatment with glucocorticoids, contributes to the well-known risk factors for developing osteopenia or osteoporosis. Only a few studies have followed JDM patients into adulthood.

## Objectives

The objective of the present study was to investigate the long-term outcome on bone mineral status in a Danish cohort of patients with JDM.

## Methods

A total of 49 patients with JDM diagnosed between 1976 and 2005 were investigated. The female/male ratio was 2.5. The mean age at disease onset was 7 years (range 1.5-16 years) and the mean disease duration was 3.7 years (range 0.7-9 years). The follow-up time ranged from 2 years to 36 years (mean 7 years). Bone mass density (BMD) (g/cm2) was determined by dual X-ray absorptiometry (DXA).

T- and Z- scores were calculated from BMD. Definition of osteopenia/osteoporosis in the adult group was according to the WHO-criteria: Osteopenia: T-score between < -1 SD and > -2.5 SD; Osteoporosis: T-score < -2.5 SD. In the children group < 20 years the guidelines from the International Society for Clinical Densitometry was used: Osteopenia: Z-score < -1 SD; Osteoporosis: Z-score < -2.0 SD.

## Results

Forty patients (75%) had a normal DXA-SCAN, 7 patients (13%) had osteopenia and 2 patients (3,8%) had osteoporosis according to the described definitions.

T-score for adults and Z-score for children were compared with normal reference values. There were no significant differences in the adult group. In children and adolescents younger than 20 years we found no significant differences in the Z-score of the whole body, but a marginally reduced Z-score of the lumbar spine.

The results are listed in Table 1.

**Table 1:** Bone Mineral Density Standard Deviation scores in 49 patients with JDM

*Score*	*N*	*Patients*	*Mean ± SD*	*Range*	P-value*	*95% CI*
***T-score***	***23***	***Adults > 20 years:*** ***- Whole body scan***	***0.43 ± 1.1***	***-1.6 - 2.6***	***0.08***	***-0.05-0.9***
	
		***Adults > 20 years:*** ***- Lumbar spine scan***	***0.26 ± 1.1***	***-1.3 - 2.1***	***0.29***	***-0.24-0.76***

***Z-score***	***26***	***Children < 20 years*** ***- Whole body scan***	***-0.02 ± 0.8***	***-2.0 - 2.0***	***0.9***	***-1.1-(-0-04)***
	
		***Children < 20 years*** ***- Lumbar spine scan***	***-0.57 ± 1.1***	***-2.7 - 1.6***	***0.04***	***-0.3 - 0.3***

*The table presents the mean T- and Z-scores with SDs and range*.

*P-values were calculated from the scores with One-Sample T-test

## Conclusion

In this JDM cohort 16.8% of the patients had osteopenia or osteoporosis. There were no significant differences in T/Z - scores between the two groups, except for a marginally reduced Z-score of the lumbar spine scan in the JDM group younger than 20 years.

The results suggest that clinical follow-up with DXA-scan is relevant for patients with JDM several years after disease remission.

## Disclosure of interest

None declared.

